# Wheat Microbiome: Structure, Dynamics, and Role in Improving Performance Under Stress Environments

**DOI:** 10.3389/fmicb.2021.821546

**Published:** 2022-01-13

**Authors:** Jian Chen, Rouhallah Sharifi, Muhammad Saad Shoaib Khan, Faisal Islam, Javaid Akhter Bhat, Ling Kui, Aasim Majeed

**Affiliations:** ^1^International Genome Center, Jiangsu University, Zhenjiang, China; ^2^Department of Plant Protection, College of Agriculture and Natural Resources, Razi University, Kermanshah, Iran; ^3^Zhejiang Key Laboratory of Crop Germplasm, Institute of Crop Science, Zhejiang University, Hangzhou, China; ^4^Guangxi Key Laboratory of Medicinal Resources Protection and Genetic Improvement, Guangxi Botanical Garden of Medicinal Plants, Nanning, China; ^5^Plant Molecular Genetics Laboratory, School of Agricultural Biotechnology, Punjab Agricultural University, Ludhiana, India

**Keywords:** wheat, microbiome, stress, rhizosphere, phylosphere

## Abstract

Wheat is an important cereal crop species consumed globally. The growing global population demands a rapid and sustainable growth of agricultural systems. The development of genetically efficient wheat varieties has solved the global demand for wheat to a greater extent. The use of chemical substances for pathogen control and chemical fertilizers for enhanced agronomic traits also proved advantageous but at the cost of environmental health. An efficient alternative environment-friendly strategy would be the use of beneficial microorganisms growing on plants, which have the potential of controlling plant pathogens as well as enhancing the host plant’s water and mineral availability and absorption along with conferring tolerance to different stresses. Therefore, a thorough understanding of plant-microbe interaction, identification of beneficial microbes and their roles, and finally harnessing their beneficial functions to enhance sustainable agriculture without altering the environmental quality is appealing. The wheat microbiome shows prominent variations with the developmental stage, tissue type, environmental conditions, genotype, and age of the plant. A diverse array of bacterial and fungal classes, genera, and species was found to be associated with stems, leaves, roots, seeds, spikes, and rhizospheres, etc., which play a beneficial role in wheat. Harnessing the beneficial aspect of these microbes is a promising method for enhancing the performance of wheat under different environmental stresses. This review focuses on the microbiomes associated with wheat, their spatio-temporal dynamics, and their involvement in mitigating biotic and abiotic stresses.

## Introduction

Modern agriculture is a result of the domestication of wild crops by humans over 1000s of years. The integrated system of agriculture is supporting the ever-growing world population. Wheat is one of the major crops, occupying around 17% of the global cultivated area and providing food for about 35% of the world’s population (Laino et al., 2015). To meet the global calorie requirement by ever-increasing human population, there is a dire need to increase wheat production by 11% till 2026 with only 1.8% increase in cultivated land (Kavamura et al., 2021). Therefore, the agricultural system has to be sustainable, and its sustainability has to be intensified, so as to make productive gains from important crops. The substantiality of the modern agricultural system has kept pace with the ever-increasing global food demands. The development of genetically improved varieties with increased yield, tolerance to biotic and abiotic stresses, improved nutrient use efficiency as well as the development of new bio-fertilizers have ensured the intensification of agricultural sustainability. However, control of biotic pathogens, and weeds, etc., is still dominated by the overuse of environmentally hazardous chemicals, whereas the enhancement of agronomic traits, especially yield, is dominated by the overuse of chemical fertilizers, which alter soil and water properties. One promising way of improving the performance of crops in terms of yield, tolerance to biotic and abiotic factors without altering the environment, is to take advantage of beneficial microorganisms present in the above and below-ground parts of plants. Both above and below-ground parts of plants are colonized with a myriad of microorganisms, many of which interact beneficially with plant to enhance their efficiency.

The ecophysiology of plant-microbe interaction is very complicated and interwoven. Therefore, a thorough understanding of the fine-tuning and integration of multiple signals generated through plant-microbe interactions is required for sustainable crop improvement. Under the natural environment, plants are exposed to a myriad of biotic and abiotic stresses; therefore, the defense responses of plants are very complex. Plant-microbe interactions can result in the prioritization of certain physiological, biochemical, and molecular pathways in plants, the dissection of which requires the application of multi-omics approaches. Using genomic, transcriptomic, proteomic, and metabolomic approaches entwined with bioinformatics have been successful in addressing microbial communities and functions within a given environment at a deeper level (de Castro et al., 2013). The pathogenic fungus, *Rhizoctonia solani* anastomosis group (AG) 8 results in substantial crop losses, including wheat and barley. In the absence of resistant cultivars to this pathogen, biological disease suppression may act as an impressive control mechanism. A thorough investigation of taxonomic and functional characteristics of the soil microbiome is therefore required to decipher the potential biocontrol agents. Through transcriptomic analysis of wheat plants grown in fields with suppressive and non-suppressive capacity against *R. solani*, Hayden et al. (2018) observed *Arthrobacter* spp. and *Pseudomonas* spp. as dominant taxa in the non-suppressive samples and *Stenotrophomonas* spp. and *Buttiauxella* spp. as dominant taxa in the suppressive samples. A higher expression of polyketide cyclase, many cold shock proteins, and a terpenoid biosynthesis backbone gene was observed in the suppressive samples, whereas the non-suppressive samples exhibited relatively greater expression of certain antibiotic genes and genes involved in mitigating oxidative damage (Hayden et al., 2018). Thus, the transcriptomic approaches have the ability to disentangle the molecular interplay of plant-microbe-pathogen interactions, the ultimate goal of which is to identify and promote the beneficial rhizosphere microbes to reduce pathogenic infections. Similarly, the meta-proteomic and metabolomic approaches have the potential to elucidate the important inter-links in plant-microbe interactions.

Recent research has witnessed the potential of microbiome organisms in fulfilling the sustainability goals without harming or altering the physio-chemical and physiological profile of the soil, water, or air. Plant microbiome helps the host species to mitigate both biotic and abiotic stress; therefore, a deeper understanding of how such microbes interact with host plants is required. The chief strategies used by microbes that can help host plants to counter stress include, but not limited to (a) resource competition with potential harmful pathogens to mitigate biotic stress, (b) inhibition of growth and development of pathogens by secretion of certain chemical substances, (c) induction of systemic acquired resistance (SAR) in host species, (d) chelation of salts, toxic and/or heavy metals through secretion of cheating agents to counter salt and heavy metal stress, (e) modulation of several gene expression modules and physiological processes to enable the host species withstand drought and temperature stress, etc. In addition, the host defense response in turn dictates the microbial community structure (Jones et al., 2019). The microbes may produce secondary metabolites, which have suppressive action on host pathogens or exhibit resource competition with the host pathogens to protect the host (Teixeira et al., 2019). A myriad of studies have confirmed the beneficial role of microbiome organisms in mitigation of biotic and abiotic stresses, and enhancement of agronomic traits of many crop species. Therefore, a better understanding of microbial profiles associated with crop species is of fundamental importance to sustainable and eco-friendly agriculture. In addition, much attention is needed to elucidate the molecular and physiological details as well as the regulation involved in such plant-microbe interactions. This review focuses on the wheat microbiome composition, how its structure changes spatially and temporally, and more importantly, the beneficial roles played by the wheat microbiome in enhancing its performance under drought, salinity, temperature, and metal toxicity stress.

## Overview of the Wheat Microbiome

**FIGURE 1:**
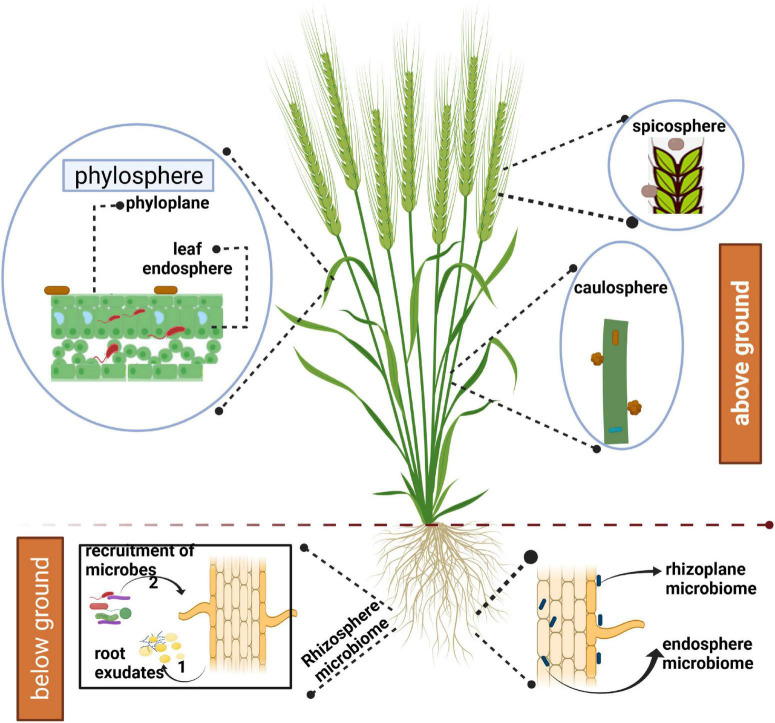
Representation of microbiomes associated with above- and below-ground parts of wheat. The figure was created with BioRender.com.

The advancement of sequencing technologies has greatly facilitated the profiling of environmental samples through metagenomic and metatranscriptomic approaches. Using amplicon sequencing, both composition and functions of microbes associated with crop species can be evaluated at relative ease now. It is possible to ascertain the effect of different factors affecting microbial communities associated with host plants in both above-ground and below-ground niches (Figure 1). The above-ground plant parts include phyllosphere (leaves), caulosphere (stems), inflorescences, and seeds. The term spicosphere was coined by Kavamura et al. (2021) to represent the niche around the spikes, as the later hosts diverse array of pathogenic and beneficial microorganisms. The below-ground niche includes rhizoplane (surface of plant roots) and rhizosphere (the soil in the vicinity of plant roots that is influenced by host plants through root exudation) (Kavamura et al., 2021). Further, microorganisms can live as endophytes in both above and below-ground plant parts. The term spermosphere describes the zone surrounding the germinating seeds (Nelson, 2004).

Although the endophytic microbes remain associated with host species throughout their life cycle, the actual community structure is governed by the tissue type. The underground plant parts harbor higher endophytic species than the above-ground parts. Most often, the endophytes are beneficial to host species as they can stimulate growth, provide protection against environmental stress, enhance nutrient absorption, and inhibit pathogenic microbes. In order to estimate the community structure and diversity of endophytic microbes from aerial and underground parts of wheat, Comby et al. (2016) observed Ascomycota (mostly Sordariomycetes or Dothideomycetes) to be the most dominant fungal phyla followed by Basidiomycota (with dominance by Agaricomycetes in which Polyporales and Russulales were top orders), and Zygomycota. The dominant bacterial classes were Gammaproteobacteia of the Proteobacteria and Bacilli of the Firmicutes. Further, the authors observed three indicator species (*Bacillus megaterium, Microdochium bolleyi*, and *Gaeumannomyces graminis*) characteristic to roots and seven indicator species characteristics to aerial parts (*Alternaria infectoria, Didymella exitialis, Epicoccum nigrum, Erwinia aphidicola, Paenibacillus hordei, Fusarium graminearum*, and *Aureobasidium protae*). Moreover, there were considerable variations in microbiome profiles with developmental stages of wheat plants (Comby et al., 2016). The *Erwinia Paenibacillus* and *Paenibacillus* were the most dominant genera associated with the wheat seeds (Robinson et al., 2016b). Further, Proteobacteria were the most prevalent endophytes in roots and Firmicutes and Actinobacteria in shoots (Robinson et al., 2016a). *Pseudomonas*, *Janthinobacterium*, and *Flavobacterium* were dominant endophytic genera in *T. aestivum* with the dominance of *Pseudomonas* and *Janthinobacterium* increasing from roots, leaves, and coleoptiles. These tissues also hosted six subdominant bacterial genera viz *Variovorax*, *Herbaspirillum*, *Caulobacter*, *Cryobacterium*, *Paenibacillus*, and *Acidovorax*. In addition to the *Pseudomonas*, *Janthinobacterium*, and *Flavobacterium*, the *Triticum spelta* also contains *Pedobacter* as a dominant genus and *Duganella, Ochrobactrum, Taibaiella*, *Rhodococcus*, *Dyadobacter*, *Mucilaginibacter*, and *Staphylococcus* as the subdominant endophytic genera in roots, leaves, and coleoptiles. Therefore, besides the three common core genera, *Pseudomonas*, *Janthinobacterium*, and *Flavobacterium* in both cultivars, the study established *Paenibacillus* as a core genus specific to *T. aestivum* and *Pedobacter* and *Duganella* as the core genera specific to *T. spelta* (Kuźniar et al., 2020). Most typical wheat endophytic species include *Achromobacter piechaudii, A. xylosoxidans, D. acidovorans, Pseudomonas monteilii, Acinetobacter lwoffii, Staphylococcus epidermis, Delftia lacustris, Ochrobactrum intermedium, Pantoea dispersa, P. eucalypti, Variovorax soli*, and *Serratia* (Mahapatra et al., 2020).

Apart from the endophytes, the microbes grow externally on the caulosphere, phyllosphere, spicosphere, rhizoplane, rhizosphere, and spermosphere of the wheat. There existed a higher fungal community abundance and diversity in wheat tissues, including roots, spikes, first stem under the ear, and stem base as well as wheat rhizosphere. Wheat varieties resistant to *Tilletia controversa*, a wheat dwarf blunt pathogen, showed greater abundance of Sordariomycetes and Mortierellomycetes, whereas the susceptible varieties exhibited higher abundances of Dothideomycetes and Bacteroidia. In contrast to other tissues, the ear and the first stem under the ear revealed greater abundances of Chloroflexi, Verrucomicrobia, Bacteroidetes, Acidobacteria, and Gemmatimonadetes. Further, *Chryseobacterium* and *Massilia*, and *Nocardioides* and *Pseudomonas* were abundant in wheat dwarf blunt infected resistant and susceptible varieties, respectively. *Curtobacterium*, *Planococcus*, *Pantoea*, and *Exiguobacterium* showed dominance in both resistant and susceptible varieties (Xu et al., 2021). The phyllospheric microorganisms can tolerate extreme temperatures as well as substantial UV radiations, so they are a class of extremophiles. The survival and proliferation of the leaf microbes largely is determined by leaf exudates like amino acids, glucose, fructose, and sucrose. Chief phyllospheric microbes include *Achromobacter, Agrobacterium, Lysinibacillus, Corynebacterium, Haemophilus, Pantoea, Alcaligenes, Streptomyces, Paenibacillus, Methylobacterium, Stenotrophomonas, Arthrobacter, Bacillus, Azotobacter, Enterobacter, Micrococcus, Brevundimonas, Micrococcus, Micromonospora, Pseudomonas*, and *Psychrobacter* (Mahapatra et al., 2020). These phyllospheric microbes mediate a variety of physiological and defense responses in wheat. Due to their close association with roots and soil, the rhizospheric microbiomes play an important role in the growth of the plant as they help the plants in nutrient and water uptake, etc. Most typical rhizospheric microbial species include *B. thuringiensis, Serratia marcescens, Azotobacter tropicalis, Rhodobacter capsulatus, Pseudomonas extremorientalis, Rhodobacter sphaeroides, P. rhizosphaerae, Arthrobacter nicotinovorans, Bacillus atrophaeus, B. horikoshii, B. mojavensis, B. siamensis, Enterobacter asburiae, Exiguobacterium acetylicum*, and *Planomicrobium okeanokoites* (Mahapatra et al., 2020).

## The Microbiome Profile of Wheat Is Spatially and Temporally Dynamic

The microbiome profile associated with host species varies with the plant part, development stage, and environmental conditions. Each stage or condition exhibits dominance of certain microbial groups that confer some positive or negative physiological impact on the host. The growth of one microbial species may alter the microbial profile associated with the host plant significantly.

### Spatial and Conditional Variation

Every species has its own peculiar microbial profile associated with its above and below-ground parts. These microbes are affected by diverse factors, thereby influencing the physiology of the host crop species. Several factors cause changes in the microbial profile associated with host species. Most common factors include anthropogenic (fungicides, insecticides, fertilization, landuse, tillage, crop rotation, and irrigation, etc.), edaphic (soil depth, soil type, and soil physiochemical properties, etc.), environmental (biotic and abiotic stress, growing season, etc.), host genotype and growth stage. Anthropogenic factors have a relatively far-most influence on the microbial profile. Use of agrochemicals like fungicides, pesticides, insecticides, and weedicides to control host pathogens and weeds, although promising, has a disadvantage of environmental pollution besides significantly altering the microbes associated with host plant species. To mitigate this problem, one solution is to use environmentally and biologically less harmful agrochemicals. However, only a few such chemicals have been screened till now. The neonicotinoid insecticides and glyphosate herbicides show no or minimal negative impact on wheat rhizosphere microbial communities (Li et al., 2018; Malalgoda et al., 2020). The variations in temperature, humidity, and precipitation significantly affect wheat microbiome composition (Latz et al., 2021). Azarbad et al. (2018) observed that water regime primarily governs the bacterial and fungal community structure in wheat rhizosphere. An increase in soil moisture alters the root exudation and soil properties along with perturbation of interactions within the rhizosphere microbiome, which specifically leads to a decrease in the production of the antibiotic phenazine-1-carboxylic acid (PCA) producers (Phz^+^) *Pseudomonas* in the rhizosphere of irrigated plants (Mavrodi et al., 2018). Further, the geographic distance (Fan et al., 2017) and seasonal changes (Schlatter et al., 2019) spatially determine the wheat microbial community structure. The biodegradable plastic mulch films also influence rhizosphere bacterial community composition and structure (Qi et al., 2020). In general, inorganic nitrogen fertilizer application negatively impacts the stability of bacterial community structure along with reduction in richness and diversity and significant depletion of Acidobacteria and Planctomycetes (Kavamura et al., 2018). Tilling also reduces bacterial diversity (Yin et al., 2010). In winter wheat, the rhizoplane and root endosphere bacterial communities were influenced by management practices (conventional vs. organic), whereas tilling affected fungal communities (Hartman et al., 2018). In comparison to monoculture, which reduces the bacterial (Mayer et al., 2019) diversity, rotation of sunflower with wheat or maize showed positive impact on bacterial communities (Wen et al., 2016). In wheat, the soil properties like pH, texture, organic, and inorganic content have also been found to influence microbial community profile (Fan et al., 2017, 2018; Schlatter et al., 2019). Besides, the microbial profile also varies with soil depth with Proteobacteriota enriched in topsoil and Firmicutes and Bacteroidota enriched in subsoil (Uksa et al., 2017; Schlatter et al., 2020). The microbial load associated with the host may originate from seed-borne microbes, which colonize the developing plant. The host species also affects the overall root microbiome to a greater degree (Cordero et al., 2020). Even the different parts of the host species reveal significant variations in microbial community structure, with Proteobacteriota dominating in the root endosphere, whereas Firmicutes and Actinobacteriota were more frequent in the leaf endosphere (Robinson et al., 2016b). Environmental factors were found to affect the phylosphere microbiome more than that of the rhizosphere in wheat (Latz et al., 2021). Further, host-driven seasonal variations in the rhizosphere microbiome were observed in wheat (Donn et al., 2015). The strong evidence that host species affects the microbial profile comes from the decreased bacterial diversity and complexity across different interaction zones between microbes and host plant (bulk soil > rhizosphere soil > rhizoplane > phylloplane > root endosphere > leaf endosphere) (Xiong et al., 2021).

### Temporal Variation

Besides, there occurs a temporal variation in microbial profile associated with host plants species during different stages of development, as the life cycle moves from seed to flowering stage. This effect is more pronounced for bacterial communities than that of fungal communities (Chen et al., 2019). The bacterial rhizosphere diversity increases with age of the host species (Donn et al., 2015; Araujo et al., 2019). Similarly, the bacterial and fungal endosphere communities (Gdanetz and Trail, 2017) and the fungal phylosphere communities (Sapkota et al., 2017) increased with age. However, the relationship between the development stage of the host species and the microbial community structure is not simple and several factors render this relationship more complex. The rhizosphere bacterial richness is reduced by some fertilization regimes. Upon high nitrogen application, the diversity remained stable over time but decreased at sub-optimal nitrogen levels. However, no effect of fertilization was observed on the decreasing root and endosphere bacterial richness with the increasing age of the host (Robinson et al., 2016a). Therefore, more research is needed to disentangle the complex relationship of microbiome and wheat developmental stages.

### Variation Due to Selection and Domestication

Modern agriculture evolved as a result of careful selection and breeding of different cultivars and a rapid transition to improved genotypes with desired characters. The wheat cultivars grown today have undergone dramatic genetic, physiological, and morphological changes from their wild relatives. In this course of transition, the root architecture might have significantly changed, which may, in turn, have affected the rhizosphere microbiome. This is evidenced by differential bacterial community structures in tall and semi-dwarf varieties of wheat (Kavamura et al., 2020). Further, during this evolution, there has been a compromise in interaction with plant growth-promoting rhizobacteria (PGPR), with ancient wild wheat varieties having a greater ability to interact with PGPR (Valente et al., 2020). The domestication of crops, especially wheat, has significantly disrupted the associated microbial community. Fungal endophytes exhibit more diversity and richness in wild wheat cultivars than in cultivated ones (Sun et al., 2020). The D genome of hexapoloid wheat strongly favors Glomeromycetes and Nematoda (Tkacz et al., 2020). Root exudation chemistry and susceptibility to pathogens may be the key factors governing the differences in microbiome-based on different wheat genotypes. Further studies are required to explore the deeper insights of the relationship between wheat genotypes and microbial community profiles. The already developed highly efficient wheat cultivars shall be screened for their influence on the microbiome profile and those cultivars with minimal negative influence shall be promoted. Moreover, the breeding programs shall take into consideration the influence of changes in the genetic architecture of a species on the microbiome profile because any negative influence of the newly developed cultivars on the microbiome profile, especially the beneficial elements, would affect its overall performance.

## Microbiomes Mitigate Stress in Wheat

**FIGURE 2:**
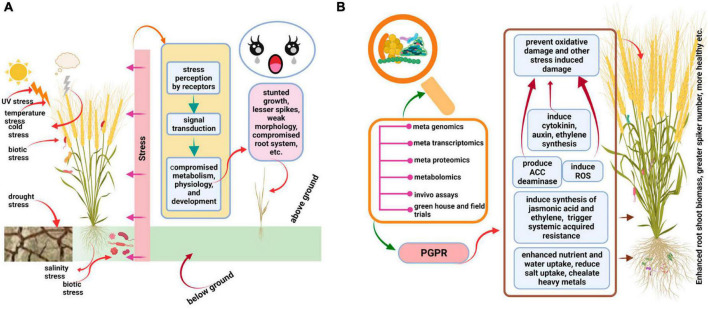
Depiction of how beneficial microbes enhance the performance of wheat under stress. **(A)** Shows that in absence of any beneficial host-microbiome interaction, wheat shows compromised growth and overall performance. **(B)** Shows that after inoculation of wheat by beneficial microbes, its root/shoot biomass and overall performance are enhanced. The figure was created with BioRender.com.

Bulk of research involving different crop species revealed that in comparison to un-inoculated plants, the plants inoculated with beneficiary microbes show better performance in terms of root/shoot growth, water and mineral absorption, yield, and resistance to biotic and abiotic stresses (Figure 2). The microbes trigger a diverse array of physiological responses in plants, including hormone production, to enhance their performance against stress. The detailed role of the wheat microbiome in ameliorating biotic and abiotic stresses is presented in the following sub-headings.

## Microbiome Assisted Resistance Against Biotic Stress

Biological control is great hope for reducing the overutilization of pesticides in agricultural soils (Köhl et al., 2019). It often involves the use of microorganisms or their metabolites that interact with either a plant or its pathogens to control the growth of the later and restrict its negative influence on the host plant. Infection of wheat plants by the fungal pathogen *Zymoseptoria tritici* resulted in suppression of the host immune system, which facilitated the colonization of other non-adapted *P. syringae* microbes on wheat (Seybold et al., 2020). Several bacterial biocontrol agents inhibit fungal pathogens. For example, they secrete lipopeptide antibiotics, phenazine derivatives, and other antifungal metabolites that negatively influence the fungal pathogen, *Fusarium graminearum* (Legrand et al., 2017). Further, the bacteria *Lysobacter enzymogenes* inhibits ceramide synthase enzyme, thereby causing degradation of the *Aspergillus* cell wall. This inhibitory effect of *L. enzymogenes* is caused due to secretion of a heat-stable antifungal factor (Li et al., 2006). The fungi, in turn, have been observed to trigger a defense response to such bacterial antibiotics. For example, *Fusarium oxysporum* produces fusaric acid to inhibit 2,4-diacetylphloroglucinol (2,4-DAPG), a broad-spectrum antibiotic produced by *Pseudomonas fluorescens* (Schouten et al., 2004) and the antifungal metabolite phenazine-1-carboxamide (PCN) by *Pseudomonas chlororaphis* (van Rij et al., 2005). Further, the beneficial bacteria also produce volatile anti-fungal metabolites, degrade the virulence factors of fungi, or even induce systemic resistance in plants against fungal pathogens (Schoonbeek et al., 2002; Effmert et al., 2012; O’Brien, 2017). Fusarium head blight (FHB) is a dangerous cereal disease caused by *Fusarium graminearum* leads to much economic losses over the world. *F. graminearum* seems to be inhibited by the bacterium *Pseudomonas piscium*. The bacteria secretes phenazine-1-carboxamide that affects the fungal histone acetyltransferase (FgGcn5), leading to deregulation of histone acetylation and growth of the fungus. Therefore, *Pseudomonas piscium* could be an important biocontrol agent of fungal wheat diseases (Chen et al., 2018).

The biocontrol agents are generally selected after their successful antagonism with the known plant pathogens *in vitro*, with in-planta screening seldom used. An in-planta study on wheat and its fungal pathogen, *F*. *graminearum* revealed several microbes, although ineffective in *in vitro* analysis but effective in in-planta screening, along with some novel strains protecting the host against fungal attack. The most important plant protectors found by the authors include *Bacillus* and *Pseudomonas*, and interestingly *Staphylococcus* (Besset-Manzoni et al., 2019). Further, the two bacterial strains, *Lactobacillus plantarum* SLG17 and *Bacillus amyloliquefaciens* FLN13 showed antimicrobial activity against *Fusarium* spp. in durum wheat and are, therefore, promising agents for the reduction of FHB index (Baffoni et al., 2015). In winter wheat, the bacterium *Sphingomonas* showed an antagonistic effect on powdery mildew and FHB. This biocontrol agent significantly reduced the population size of *Fusarium poae* and severity of infection by *Blumeria graminis* f. sp. t*ritici* (Wachowska et al., 2013). Moreover, the bacterial inocculents *Paenibacillus jamilae* and *Bacillus amyloliquefaciens* were observed to reduce (82 and 83%, respectively) soil-borne wheat diseases and significantly affect the rhizosphere microbial community structure. The beneficial rhizosphere bacteria like *Sphingomonas*, *Bacillus*, *Nocardioides*, *Rhizobium*, *Streptomyces*, *Pseudomonas*, and *Microbacteriu* and the beneficial fungal genera like *Chaetomium*, *Penicillium*, and *Humicola* were enriched, whereas the pathogenic fungal strains, *Fusarium* and *Gibberella* were restricted upon treatment of *Paenibacillus jamilae* and *Bacillus amyloliquefaciens* (Wang et al., 2021). Likewise, *Stenotrophomonas maltophilia, Bacillus cereus*, and *richoderma harzianum* showed biocontrol activity against *F. graminearum* (Dal Bello et al., 2002). The yeast isolates of the genera *Cryptococcus, Rhodotorula*, and *Saccharomyces* showed an inhibitory effect on the development of *F. sporotrichioides* colonies in wheat. Further, the bacterial isolate *Sphingomona*s inhibited the fungal pathogenic species *F. avenaceum, F. culmorum, F. tricinctum*, and *F. graminearum* (Wachowska et al., 2013).

Tan spot is the most destructive global foliar wheat disease, caused by *Pyrenophora tritici-repentis.* Using both double culture and greenhouse assays to evaluate the effect of wheat endophytes on the growth and sporulation on the tan spot pathogen, Larran et al. (2016) observed significant *in vitro* reduction of the pathogen colony diameter by *Trichoderma hamatum*, *Penicillium* sp., *Bacillus* sp., and *Paecilomyces lilacinus.* Further, *Bacillus* sp. and *Fusarium* sp. reduced the spore germination by 82 and 52%, respectively. Moreover, *T. hamatum, Chaetomium globosum* and *Fusarium* sp. were found to significantly reduce the severity of the disease on wheat leaves in green house assays, with greatest suppression by *T. hamatum* (Larran et al., 2016). The “take all” root disease in wheat, caused by an ascomycete fungus, *Gaeumannomyces graminis* affects plant growth and yield. Antagonistic assay of the bacterial isolates collected from the rhizosphere of wheat roots on *G. graminis* revealed highest inhibitory action of *Burkholderia* sp., *Pseudomonas* sp., *Bacillus subtilis*, and *Xanthomonas* sp. (Nasraoui et al., 2007). With the aim of characterizing the effect of infection with *Zymoseptoria tritici* fungus on the wheat microbial communities and to identify microorganisms interacting with it, Kerdraon et al. (2019) used meta-barcoding approach and observed significant alteration in microbial communities with some species remaining affected even after the disappearance of the pathogen. Using the pyrosequencing technique, certain microbes were found to suppress *Rhizoctonia* root rot and bare batch disease in wheat, with *Chryseobacterium* and *Pseudomonas* dominating in the rhizosphere over time (Yin et al., 2013). In order to monitor the changes in microbial communities associated with wheat, application of the biocontrol agents, *Paenibacillus fulvissimus* and *Streptomyces* spp. to *Rhizoctonia solani* and *Pythium* sp. infected soils revealed modulation of the endosphere and rhizosphere microbiomes. Further, a low impact on indigenous microbial communities, reduction in root disease, and plant growth enhancement were observed (Araujo et al., 2019, 2020).

## Microbiome Assisted Resistance Against Abiotic Stress

Among the principle factors responsible for declining agricultural produce, abiotic stress stands at the forefront. Around 64% of the land area is affected by drought, 13% by anoxia due to flood submergence, 57% by cold, 15% by acidic soils, 6% by salinity, and 9% by mineral deficiency (Cramer et al., 2011). Around 69.23% of the dryland agriculture is influenced by soil salinity, erosion, and degradation (Riadh et al., 2010). Plants possess internal metabolic re-programming and homeostatic dynamics to cope up with adverse conditions. Following the sensing of stress signal, plants trigger a protective cascade involving synthesis of phytohormones, accumulation of flavonoids and phenolics, onset of antioxidants and osmolytes, and activation of specific genes and their regulation through repression/activation of transcription factors, which ultimately confers timely defense to plants (Meena et al., 2017). The microbes associated with plants form specific interactions with their hosts to evoke local and systemic responses for providing indispensable resistance against abiotic stress. Therefore, plant-microbe interactions are viewed as a key adaptive survival strategy in abiotic stress. The stress response provoked by microbe is termed Induced Systemic Tolerance (IST). The role of microbes in mitigating various biotic stresses has been documented for many crop species (Meena et al., 2017).

### Mitigation of Drought Stress

Drought stress is one of the major concerns of agricultural losses worldwide. Maintaining increased crop productivity requires efficient low-cost technologies for abiotic stress management. Drought leads to loss of turgor, an increase in ionic concentration, and cell viscosity. Under drought, plants retain water by decreasing their water potential by making osmotic adjustments (OA) through production of osmolytes like proline, glycine betaine, polyamines, polyols, soluble sugars, and ions especially K^+^. Roots are first to sense drought signal. An improved root system in wheat is resistant to drought. In wheat, the soluble sugars contribute most to OA, and starch is degraded to soluble sugars under drought stress. Besides, increase in proline and other amino acids, reactive oxygen species (ROS), antioxidant molecules like catalase, superoxide dismutase (SOD), glutathione peroxidase (GPX), ascorbate peroxidase (APX), and glutathione reductase (GR) are triggered to mitigate drought stress. Hormones, notably abscisic acid (ABA), which causes stomatal closure and expression of ABA-responsive genes to protect the plant from water loss, cytokinin that acts antagonistically to ABA under drought, and ethylene, which at thresh hold levels under acute drought cause senescence, are also involved in drought response. The MYC/MYB, NAC, AREB/ABFs, DREB or CBF, and WRKY transcription factors also regulate drought response through expression/repression of their target genes. Further, volatile organic compounds like isoprenoids are used as a signal to communicate within the plant and with other plants and trigger a stress tolerance in these plants (Camaille et al., 2021).

Several strategies have been proposed to mitigate the adverse effects of drought stress in plants; however, most of these approaches are time-consuming, cost-intensive, and not well accepted in some regions (Wahid et al., 2007). A promising alternative approach is to induce stress tolerance in plants by using beneficial microorganisms. The plant growth-promoting rhizobacteria (PGPR) modulate growth and development to enhance stress tolerance through physiological responses in plants. Besides, PGPR also improves plant performance by causing improved soil structure and increased soil water retention. The application of PGPRs that coevolved with plant roots over millions of years under harsh environments to improve plant fitness under biotic and abiotic stresses has advantages over stress management through genetic modifications. For example the phenazine-producing bacteria grow abundantly in the rhizosphere of dryland-grown wheat. They perform better in rhizospheres with lower moisture content and are particularly abundant in drought-resistant cultivars (Mavrodi et al., 2012, 2018; Mahmoudi, 2017). The wheat seedlings exposed to water deficit but colonized by the phenazine-producing strains of *Pseudomonas* suffered less dehydration and recovered better, thereby conferring better resilience to seedlings. The inoculated seedlings showed higher growth of root system, especially tips. Thus, the phenazine-producing bacteria in the rhizosphere could be harnessed for drought stress management in wheat (Mahmoudi et al., 2019). *Burkholderia phytofirmans* strain PsJN is an extensively studied endophytic bacteria that colonize a wide variety of plants. Significant dilution of adverse effects of drought stress coupled to improvement of the photosynthetic rate, water use efficiency, chlorophyll content, grain yield, and NPK levels in grains was observed in wheat after inoculation with PsJN strain. This revealed that *B. phytofirmans* has the potential to improve the growth, physiology, and quality of wheat under drought conditions (Naveed et al., 2014). In order to determine the beneficial effect of *Azospirillum brasilense* on wheat suffering from drought during anthesis, relatively higher water content and water potential, apoplastic water fraction, and a lower cell wall modulus of elasticity were observed along with lesser yield loss in trials inoculated with *Azospirillum* than those of non-inoculated ones. Further, the grains obtained from inoculated plants exhibited significantly higher Mg, K, and Ca contents, showing that a higher growth can be promoted under drought stress in wheat through microbial associations (Creus et al., 2004). Further, the priming of wheat seedlings with the rhizosphere bacteria caused enhancement in drought tolerance and resulted in 78% greater biomass and a fivefold higher survival rate. The ROS scavenging enzyme activities increased in response to bacterial priming. Out of the seven volatile compounds emitted from the leaves of the primed seedlings, monitoring of the three viz benzaldehyde, beta-pinene, and geranyl acetone is key to characterize the efficiency of different bacterial strains in priming for drought stress resistance (Timmusk et al., 2014). Besides, inoculation of wheat plants with *Bacillus safensis* or *Ochrobactrum pseudogregnonense* (Chakraborty et al., 2013), *Azospirillum lipoferum* (Arzanesh et al., 2011), *Pantoea alhagi* (Chen et al., 2017), *Bacillus amyloliquefaciens* and *Azospirillum brasilense* (Kasim et al., 2013), *Bacillus thuringiensis* (Timmusk et al., 2014), *Burkholderia phytofirmans* (Naveed et al., 2014), *Klebsiella* sp. (Gontia-Mishra et al., 2016) trigger diverse physiological effects to enhance survival and/or yield under water deficit. Further, to mitigate water stress, *A. brasilense* (Creus et al., 2004), *B. safensis* or *O. pseudogregnonense* (Chakraborty et al., 2013) result in enhancement of osmolytes in wheat, however, inoculation of *Klebsiella* sp., although improved root and shoot growth, lowers the total soluble sugars and proline content in wheat (Gontia-Mishra et al., 2016). Moreover, the PGPRs produce their own volatile compounds like 2,3-butanediol, acetoin, or acetic acid. The acetic acid enhances the formation of biofilm by Exopolysaccharides (EPS) producing bacteria, 2,3-butanediol induces drought tolerance through stomatal closure and reduction of water loss (Camaille et al., 2021). Certain PGPRs have the ability to degrade ethylene precursor ACC into ammonium and α-ketobutyrate through the production of 1-aminocyclopropane-1-carboxylate deaminase (ACCd) enzyme. This reduces the ethylene level in the plant and reduces adverse effects of so-called stress ethylene. ACCd is produced in many PGPR bacterial genera like *Pseudomonas, Bacillus, Rhizobium, Sinorhizobium, Variovorax, Burkholderia* or *Azospirillum* (Camaille et al., 2021). Wheat seedling primed with ACCd producing *Bacillus subtilis* (Barnawal et al., 2017), *Klebsiella* sp. (Gontia-Mishra et al., 2016) exhibited reduced ACC content and better photosynthetic efficiency, root-shoot growth under drought stress. PGPRs can trigger the production of phytohormones, especially auxins in plants, which could modify RSA. Inoculation of wheat seedlings with IAA-producing *Klebsiella* sp. (Gontia-Mishra et al., 2016), *Azospirillum* sp. (Arzanesh et al., 2011), *Bacillus, Enterobacter, Moraxella*, and *Pseudomonas* (Raheem et al., 2018), *B*. *subtilis* (Barnawal et al., 2017) enhances root number and length, photosynthetic efficiency under drought stress, which allows better assimilation of water and nutrients (Barnawal et al., 2019). Barnawal et al. (2017) observed a 30% reduced ABA level in wheat seedlings primed with PGPRs along with 28% increase in shoot dry weight and 17% increase in root dry weight. Several bacterial attributes which are involved in promoting drought tolerance include the ability to produce 1-aminocyclopropane-1-carboxylate deaminase (ACCd), indole-3-acetic acid (IAA) and siderophores. ACCd acts by preventing ethylene from reaching inhibitory levels to allow proper root growth under water deficit, IAA regulates stomatal aperture and enhances root and shoot growth under drought, siderophores allow proper nutrient cycling under drought (Hone et al., 2021).

The microbial species associated with wheat also enhance drought resistance through the enhancement of antioxidant response. They increase the activity of ROS-scavenging enzymes like GR, SOD, CAT, peroxidase, APX, and GPX, etc (Chakraborty et al., 2013; Timmusk et al., 2014). Kasim et al. (2013), however, obtained decreased activity of ROS-scavenging enzymes APX and dehydroascorbate reductase upon inoculation of wheat seedlings with *Bacillus amyloliquefaciens* and *Azospirillum brasilense*. In this case, the stress mitigation activity of the microbes may be associated with a different mode like the production of ACCd or IAA (Kasim et al., 2013). The EPS is a complex mixture of biomolecules produced by bacterial species. EPS-producing bacterial in the rhizosphere lead to a better soil aggregation around the roots and more efficient water and nutrient flux toward the plant roots. They confer several beneficial properties to the host plant, including tolerance against drought stress. *B. thuringiensis*, when inoculated with wheat, produces biofilm around roots which leads to 2–3 fold more soil aggregation around roots, 63% enhancement in water use effeciency, and higher survival rate under drought stress (Timmusk et al., 2014). Similar results were obtained by inoculation of wheat with *Klebsiella* sp. (Gontia-Mishra et al., 2016). Seed microbes play a vital role in the transmission of microbes between plant generations. Seed microbes are the initial colonizers of plant tissues and are believed to shape the overall plant microbiome composition, therefore have a competitive advantage over microbes recruited from soil and root. The use of metagenomic and culture-based methods for profiling and characterizing the seed microbiome structure in drought-tolerant and susceptible wheat cultivars have shown growth promotion of wheat by *Curtobacterium flaccumfaciens* and *Arthrobacter* sp. under drought conditions. The beneficial seed microbes were line-specific and responsive to environmental stress. The study indicated that seeds collected from stressed plants form an important resource to identify microbes with growth-promoting activity (Hone et al., 2021).

### Mitigation of Temperature Stress

Fungal root endophytes are taxonomically, ecologically, and physiologically poorly understood in comparison to their phylosphere counterparts. Fungal endophytes belonging to Ascomycota and Basidiomycota are involved in thermo- and drought tolerance in different plant species (Redman et al., 2002; Márquez et al., 2007; Sherameti et al., 2008; Sun et al., 2010). The endophytic fungi even form symbiotic associations (called mycovitality) with seeds (Vujanovic and Vujanovic, 2007). A close relationship between the endophytic fungal compartmentalization and plant health was observed in wheat (Abdellatif et al., 2009). Endophytic Ascomycetes improve seed germination in wheat under heat and drought stress (Hubbard et al., 2012). Evaluation of the impact of fungal endophytes on the growth, ecophysiological, and reproductive success of heat and drought-stressed wheat revealed that the photosynthetic effeciency, plant height, seed weight showed a general improvement in plants colonized by endophytic fungi as compared to endophyte-free plants. The endophytes promoted heat stress in wheat through the reduction in photosynthetic stress. They enhanced percentage germination and decreased time to 50% germination, proving their capacity to enhance heat and drought stress in parental plants and second-generation seeds via mycovitality (Hubbard et al., 2014). Evaluation of plant-growth-promoting effects and nutrient uptake by PGPR isolated from rhizosphere, phyllosphere and soil in winter wheat showed that both soil type and temperature influence the growth-promoting effects of the bacteria. The root and shoot growth were enhanced under low temperature of 16°C when wheat plants were inoculated by *Pseudomonas fluorescens*, *Pantoea agglomerans*, and *Mycobacterium sp.*, whereas *Mycobacterium phlei* and *Mycoplana bullata* improved the root and shoot growth under nutrient-poor medium. Besides, all the bacteria were found to enhance NPK content of plants (Egamberdiyeva and Höflich, 2003). Using 16S rRNA sequencing, a bacteria collected from Amarnath soils of North-Western Himalayas of India, was identified as *Pseudomonas lurida*, which possessed unique properties, including growth at as low as 4°C. The bacteria have the ability to produce IAA and solubilize phosphate even at 4°C. Inoculation of this bacterium with wheat seeds showed growth-promoting ability. The seed germination percentage, shoot and root lengths were significantly increased in inoculated plants as compared to non-inoculated ones. The strain produced siderophores at mesophilic and cold temperatures, which in low nutrient environments secrete ion-binding ligands, especially iron-binding (siderophores) that bind to ferric iron and make it available to the host microorganisms. All these properties can prove beneficial in wheat breeding in cold environments (Mishra et al., 2009). Based on the evaluation of 23 parameters, investigation of cold response in wheat seedlings inoculated with *Pseudomonas* strains under greenhouse conditions showed that the un-inoculated plants were under cold stress and that eight strains alleviated cold stress in wheat. Inoculation significantly improved chlorophyll, anthocyanin, proline, phenolic, and starch contents along with physiologically available Fe, proteins, and cold tolerance amino acids. Reduced electrolyte leakage and Na^+^/K^+^ ratio were also observed in inoculated plants, proving the efficacy of Pseudomonas strains in mitigating cold stress, and improving the performance of wheat in cold environments (Mishra et al., 2011).

### Mitigation of Salinity Stress

Salinity affects around one-third of the world’s arable land resources (Qadir et al., 2000). The important attributes which determine salt tolerance of plants depend on the restricted/controlled uptake of Na^+^ and Cl^–^, greater intake of K^+^ and NO3^–^, and preferential uptake of K^+^ over Na^+^ by roots (Jeschke and Wolf, 1988). To adapt to high salt concentrations, plants use following strategies; (a) activation of Na^+^ efflux, (b) prevention of Na^+^ influx, and (c) Na^+^ compartmentalization in vacuoles (Rajendran et al., 2009). The Na^+^/H^+^ antiporters (SOS1 and NHX1) maintain the optimum ionic concentrations in the cytoplasm to avoid toxicity due to Na^+^. Plasma membrane located SOS1 extrudes Na^+^ from cytoplasm to apoplast, whereas the tonoplast located NHX1 pumps Na^+^ from cytoplasm to vacuoles (Gaxiola et al., 1999; Shi et al., 2000). Both transporters require energy, which is provided through proton motive force generated by H^+^-ATPase. In any salt tolerance, the activity and expression of these transporters play a central role in maintaining the optimal concentration of Na^+^ in the cytoplasm to avoid salinity-induced damage to the cell. Salinity stress often induces overproduction of ROS like hydroxyl radical (OH^⋅^), single oxygen (^1^O_2_), superoxide anion (O_2_^⋅—^), and hydrogen peroxide (H_2_O_2_). These ROS species are very dangerous as they deteriorate membrane structure and alter its permeability. They, therefore, must be immediately scavenged to avoid oxidative damage. Plants exhibit enzymatic and non-enzymatic antioxidant defense systems to neutralize ROS-induced oxidative damage. Chelation of cations or their compartmental sequestration is yet another strategy adapted by plants to counter salt stress. Ethylene is an important plant growth regulator controlling many important developmental processes like seed germination, root hair development and elongation, fruit ripening, leaf abscission, and organ senescence (Ahmad et al., 2011). However, under stress, the ethylene is accumulated in higher detrimental concentrations, which can inhibit plant growth (Erice et al., 2017). Therefore, regulation of ethylene concentration to optimal levels during stress is of utmost importance to avoid ethylene-induced detrimental effects. The level of ethylene can be checked through regulation of ACC deaminase that cleaves ethylene precursor ACC to ammonia and α-ketobutyrate (Kumar et al., 2020). Therefore, role of ACC deaminase in salt and other type of stresses is crucial. Accumulation of compatible solutes such as sugars and proline in root vacuoles, as well as of Ca^2+^ and K^+^ also confer salinity tolerance to plants.

Bacterial EPS have the ability to bind cations, including Na^+^ (Geddie and Sutherland, 1993). Therefore, the abundance of such bacterial species in the rhizosphere would significantly lock up the Na^+^ ions and confer beneficial advantage to plants under salinity stress. Inoculation of wheat seedlings grown in a moderately saline soil by EPS-producing bacteria like *Aeromonas hydrophila/caviae* (strain MAS-765), *Bacillus insolitus* (strain MAS17), and three *Bacillus* sp. strains (MAS617, MAS620, and MAS820) showed an increase in root and shoot dry matter and reduced Na^+^ uptake by roots, which indicate that the inoculated plants performed better under salt stress (Ashraf et al., 2004). The utilization of plant root-associated stress-tolerant microbial species has the potential to improve soil fertility and plant resistance toward adverse environmental conditions (Wu et al., 2009). Consequently, the microbial species dwelling in high saline environment can provide salt tolerance to host species. The high saline habitat-dwelling microbial species like *Bacillus pumilus*, *Pseudomonas mendocina*, *Arthrobacter* sp., *Halomonas* sp., and *Nitrinicola lacisaponensis* have a growth-promoting effect on wheat grown in saline soils. Besides improving certain growth parameters like root/shoot length, root-shoot biomass ratio, chlorophyll, carotenoid, and protein content, the wheat plants inoculated by these species also accumulated higher phenolics, flavonoids, and IAA in the rhizosphere, as well as promoted the overall plant growth in the saline soils (Tiwari et al., 2011). Halotolerant bacterial strains can survive under high salinity, they overcome the adverse effects of high salt by compatible solute accumulation, production of extracellular proteases, and activation of Na^+^/H^+^ antiporters, etc. Many of the halotolerant microbes possess PGPR properties, therefore can be harnessed to mitigate salt stress in different crop species. In addition to the coating of wheat seeds by phytohormone-rich extract filtered from the bacterial culture, the inoculation of wheat seedlings with a halotolerant methylotrophic *Nocardioides* sp. under saline conditions revealed improved germination in the primed seeds, enhanced growth, protein content, and activity of SOD, CAT, APX, and peroxidase. Further, over-expression of defense-related genes was observed in the seedlings, indicating the potential of the bacterium in salt stress (Meena et al., 2020). Screening of halotolerant bacterial strains from the saline habitats revealed *Hallobacillus* sp. and *Bacillus halodenitrificans* as the potential microbes with PGPR properties under salt stress in wheat (Ramadoss et al., 2013). None of the bacteria, however, had ACC deaminase activity. As IAA may promote root growth by stimulating cell elongation or cell division, evaluation for IAA accumulation revealed that this stress mitigating property on wheat was due to IAA production only in the case of *Hallobacillus* sp. Inoculation of the carotenoid-producing halotolerant bacteria *Dietzia natronolimnaea* improved growth in terms of dry weight and height, photosynthetic pigments, CAT, and peroxidase in wheat under salt stress. Further, modulation of the transcriptional machinery to induce salt tolerance in wheat was observed. The ABA-responsive genes (*ABARE*) and *TaOPR1*, the Salt Overly Sensitive (SOS) genes (*SOS1* and *SOS4*), the transcription factors (*TaWRKY10*, *TaWRKY17*, and *TaMYB33*), the ion transporter genes *TaHKT*, *TaNHX*, *TaHAK*, and the antioxidant genes *POD, CAT, APX, GR, GPX, MnSOD* were over-expressed under salt stress in *D. natronolimnaea* inoculated plants as compared to non-inoculated ones. This indicates that the halotolerant *D. natronolimnaea* induces salinity tolerance through a complex intermingled process involving modulation of ABA-signaling, SOS pathway, ion transporters, and antioxidant machinery (Bharti et al., 2016). The microbial species also mitigate salt stress through up-regulation of antioxidant defense response. Wheat seedlings inoculated with *Piriformospora indica* under salinity stress showed lower lipid peroxidation, relative membrane permeability, and lipoxygenase enzyme (LOX) activity along with the higher accumulation of proline, α-tocopherol, and carotenoids and enhanced activity of SOD, CAT, and APX compared to un-inoculated plants (Singh and Tiwari, 2021).

Besides the production of plant growth regulators like auxins, cytokinins, and gibberellins, PGPRs also have the ability to bring ethylene levels to normal under stress through the production of ACC deaminase that cleaves ethylene precursor ACC to ammonia and α-ketobutyrate (Kumar et al., 2020). Most of the ACC deaminase-producing PGPRs modify root number and area to enhance nutrient uptake from stressed soils. Under salt and drought stress, ABA acts by causing accumulation of the compatible solutes such as sugars and proline in root vacuoles, as well as of Ca^2+^ and K^+^, which mitigate the effects of high salinity, and also cause stomatal closure to prevent excessive water loss to mitigate drought effects. The uptake and accumulation of essential nutrients like NPK and H_2_O are reduced under salinity stress. Different microbial strains have been shown to enhance the P and K uptake under salt stress (Mayak et al., 2004; Kang et al., 2014). PGPRs also enhance the availability of mineral elements like Cu, Fe, Mn, and Zn, etc., to plants by chelation and acidification of soil (Kumar et al., 2020). The bacterial siderophores have a higher affinity toward Fe than siderophores produced by plants. *Invitro* assessment of the rhizosphere bacteria *Pseudomonas fluorescens*, *Serratia liquefaciens*, *Bacillus subtilis*, and *Bacillus megaterium* revealed their salt tolerance up to 3% salinity levels and ability to produce IAA. *P. fluorescens* and *B. megaterium* also showed ACC deaminase activity. *Invivo* assessment of the salt stress mitigating capacity of the later two revealed improved wheat growth under salinity stress (Fathalla and Abd El-Mageed, 2020).

### Mitigation of Metal Toxicity

Heavy metals like lead (Pb), arsenic (As), and Nickel (Ni), etc., especially in soil, pose another stress threat to plants. Once accumulated in soils, they affect the soil dynamics, soil microbial composition and functions, which ultimately leads to loss of soil fertility, thereby crop production. Like other stress types, heavy metal toxicity induces oxidative damage by producing more ROS, alters enzymatic activities and water and mineral transport, impairs growth and developmental processes, decreases the performance of the plant, and causes yield losses. Plantation of species with phytoremediation potential is one of the strategies to manage heavy metal toxicity. An alternative strategy for managing soils polluted by heavy metals is to utilize heavy metal tolerant PGPRs (Liu et al., 2015). As biofertilizers, the heavy metal tolerant microbes detoxify heavy metals by altering/limiting the bioavailability of metals through acidification, chelation, precipitation, exclusion, sequestration or transformation to less toxic forms (Dixit et al., 2015; Mishra et al., 2017). Further, the metal tolerant microbes release metallothioneins (MTs) and phytochelatins (PCs) that chelate metals to confer defense against heavy metals (Jan et al., 2014). *Bacillus subtilis* showed tolerance against Ni and Pb. The oxidative damage and growth inhibition were suppressed when the wheat plants under Ni and Pb stress were inoculated with *B. subtilis*. Shoot length and biomass and grain yield were improved. The growth-promoting effect of *B. subtilis* was due to IAA generation, which accelerates cell division and enlargement, root elongation, and apical dominance in wheat. Further, small amounts of siderophores in liquid medium only were produced by *B. subtilis*, which could confer stress tolerance to wheat. Moreover, the bacteria also showed ACC deaminase activity, which although was reduced by increasing Ni and Pb concentrations (Rizvi et al., 2019).

## Host-Mediated Microbiome Engineering

**FIGURE 3:**
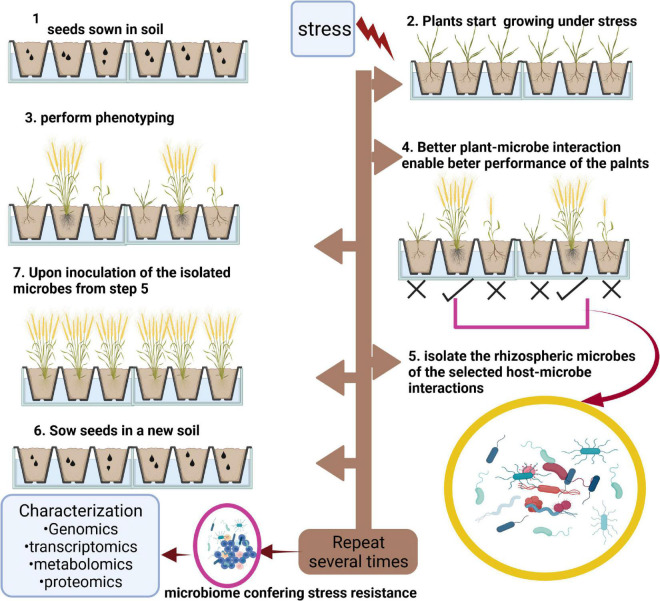
Graphical representation of host-mediated microbiome engineering. The figure shows that under stress, some genotypes perform better than others (step 4), isolation (step 5), and inoculation of the microbes associated with these genotypes (step 6) with new soil result in better performance of plants. Repetition of steps 2–7 would result in a microbiome having the property to confer stress resistance to host species. The figure was created with BioRender.com.

In a generalized fashion, the host-mediated microbiome engineering (HMME) is a technique to selectively pick the rhizosphere microbiome of the most tolerant plants after induced stress, inoculate a fresh sterile medium with the selected rhizomicrobiome followed by planting of new germplasm in the inoculated medium (Figure 3). After several cycles with the same host, a microbiome is obtained that confers beneficial interactions to the host. This microbiome, which is now established for its beneficial role under stress, can be characterized in detail using different omics technologies. Therefore, HMME is a host-centric selection process of microbiomes at the community level (Swenson et al., 2000). Both ecological and evolutionary processes are involved in the change in microbiome during HMME process (Mueller and Sachs, 2015). A good amount of evidence has accumulated in recent years acknowledging the potential of HMME in conferring beneficial adaptations to host plants. For example, HMME selected microbiomes provided enhanced growth under less favorable soil pH (Swenson et al., 2000) and altered flowering onset and leaf biomass (Panke-Buisse et al., 2014, 2017) in *Arabidopsis thaliana.* With the aim to improve seedling establishment under extreme drought in wheat, Jochum et al. (2019) used delayed onset of seedling water deficit stress symptoms phenotype to select microbiomes that could provide beneficial interaction to host and enable its better withstand and survival. Following six rounds of artificial selection, the research group obtained a microbial community that not only delayed the stress symptoms in seedlings as long as 5 days but also improved the overall biomass, root length, surface area, and dry weight. This beneficial effect was completely lost upon autoclaving of the medium, indicating the true relationship of the artificially selected microbiome with the host. Taxonomic investigation of the selected microbiome indicated Proteobacteria and Bacteroidetes as the most abundant phyla followed by Actinobacteria, and Firmicutes. Gemmatimonadetes was the least abundant phyla followed by Cyanobacteria in the selected microbiome. Among the dominant classes were Betaproteobacteria followed by Gamma-, Delta- and Alphaproteobacteria (Jochum et al., 2019 for image). HMME selected microbiome was also found to provide salt tolerance to *Brachypodium distachyon* (Mueller et al., 2016). Using HMME, specific microbiomes can be engineered to aid wheat and other crop species in mitigating salt, drought, heat, and other types of biotic stresses.

## Future Perspectives

It is now well established that microbiomes mediate diverse beneficial functional roles in the host species. Harnessing these beneficial interactions for sustainable agriculture provides several advantages over other approaches of crop improvement, especially the use of excessive fertilizers, pesticides, and insecticides, etc., for enhancement of yield and crop protection. These chemicals alter the soil microbiome and in certain cases restrict the beneficial microbial species. The details about the effect of these factors on the dynamics of microbial community structure in most crop species are very scarce. Therefore, an ample research shall be focused to dissect how fertilizers, pesticides, and insecticides etc., regulate the beneficial microbes of the host species, especially wheat. Evaluation of trade off between the use of these chemicals and the negative impact of reduction of beneficial microbes by these chemicals on the overall plant performance would provide a useful guide to use these chemicals at optimal quantities, at which there is minimal negative effect on the beneficial microbes. Further, the development of elite cultivars through breeding programmes also influence the host microbiome profile. There is a dire need to generate a robust and elaborate database of cultivar type and associated microbiomes, which would give us an idea about the missing beneficial microbes for each cultivar. Search for alternative microbes providing similar beneficial roles and hosted by the cultivar would suffice for the missing cultivar specific beneficial microbes. The crop improvement programs, during the development of elite cultivars, shall include the assessment of dynamic changes on the microbiome profile as an important parameter in the list of other commonly used screening traits.

## Author Contributions

AM drew the figures. All authors contributed to writing of the manuscript.

## Conflict of Interest

The authors declare that the research was conducted in the absence of any commercial or financial relationships that could be construed as a potential conflict of interest.

## Publisher’s Note

All claims expressed in this article are solely those of the authors and do not necessarily represent those of their affiliated organizations, or those of the publisher, the editors and the reviewers. Any product that may be evaluated in this article, or claim that may be made by its manufacturer, is not guaranteed or endorsed by the publisher.
