# Corrigendum: Wheat Microbiome: Structure, Dynamics, and Role in Improving Performance Under Stress Environments

**DOI:** 10.3389/fmicb.2022.940368

**Published:** 2022-05-31

**Authors:** Jian Chen, Rouhallah Sharifi, Muhammad Saad Shoaib Khan, Faisal Islam, Javaid Akhter Bhat, Ling Kui, Aasim Majeed

**Affiliations:** ^1^International Genome Center, Jiangsu University, Zhenjiang, China; ^2^Department of Plant Protection, College of Agriculture and Natural Resources, Razi University, Kermanshah, Iran; ^3^Zhejiang Key Laboratory of Crop Germplasm, Institute of Crop Science, Zhejiang University, Hangzhou, China; ^4^Guangxi Key Laboratory of Medicinal Resources Protection and Genetic Improvement, Guangxi Botanical Garden of Medicinal Plants, Nanning, China; ^5^Plant Molecular Genetics Laboratory, School of Agricultural Biotechnology, Punjab Agricultural University, Ludhiana, India

**Keywords:** wheat, microbiome, stress, rhizosphere, phylosphere

In the original article, there was a typo error for two words, suppressed and non-suppressed, which were used in place of each other in the section Introduction. Further, an accidental deletion of a part of that sentence happened during editing of the final version of the manuscript before communicating to the Journal, which changed the meaning of the sentence. A correction has been made to **Introduction**, paragraph two. The corrected paragraph appears below.

“The ecophysiology of plant-microbe interaction is very complicated and interwoven. Therefore, a thorough understanding of the fine-tuning and integration of multiple signals generated through plant-microbe interactions is required for sustainable crop improvement. Under the natural environment, plants are exposed to a myriad of biotic and abiotic stresses; therefore, the defense responses of plants are very complex. Plant-microbe interactions can result in the prioritization of certain physiological, biochemical, and molecular pathways in plants, the dissection of which requires the application of multi-omics approaches. Using genomic, transcriptomic, proteomic, and metabolomic approaches entwined with bioinformatics have been successful in addressing microbial communities and functions within a given environment at a deeper level (de Castro et al., 2013). The pathogenic fungus, *Rhizoctonia solani* anastomosis group (AG) 8 results in substantial crop losses, including wheat and barley. In the absence of resistant cultivars to this pathogen, biological disease suppression may act as an impressive control mechanism. A thorough investigation of taxonomic and functional characteristics of the soil microbiome is therefore required to decipher the potential biocontrol agents. Through transcriptomic analysis of wheat plants grown in fields with suppressive and non-suppressive capacity against *R. solani*, Hayden et al. (2018) observed *Arthrobacter* spp. and *Pseudomonas* spp. as dominant taxa in the non-suppressive samples and *Stenotrophomonas* spp. and *Buttiauxella* spp. as dominant taxa in the suppressive samples. A higher expression of polyketide cyclase, many cold shock proteins, and a terpenoid biosynthesis backbone gene was observed in the suppressive samples, whereas the non-suppressive samples exhibited relatively greater expression of certain antibiotic genes and genes involved in mitigating oxidative damage (Hayden et al., 2018). Thus, the transcriptomic approaches have the ability to disentangle the molecular interplay of plant-microbe-pathogen interactions, the ultimate goal of which is to identify and promote the beneficial rhizosphere microbes to reduce pathogenic infections. Similarly, the meta-proteomic and metabolomic approaches have the potential to elucidate the important inter-links in plant-microbe interactions.”

The authors apologize for this error and state that this does not change the scientific conclusions of the article in any way. The original article has been updated.

## Publisher's Note

All claims expressed in this article are solely those of the authors and do not necessarily represent those of their affiliated organizations, or those of the publisher, the editors and the reviewers. Any product that may be evaluated in this article, or claim that may be made by its manufacturer, is not guaranteed or endorsed by the publisher.
